# The effect of servitising level on firm performance of listed Chinese sporting goods manufacturing companies—With moderated mediation effect

**DOI:** 10.1371/journal.pone.0297226

**Published:** 2024-02-05

**Authors:** Laibing Lu, Wei Pan, Haixia Wang, Shaoxiong Yang, Zhiyong Liu, Qiuying Li

**Affiliations:** 1 School of Physical Education and Sport Science, Fujian Normal University, Fuzhou, China; 2 College of Physical Education,Handan University, Handan, China; 3 Department of Public Sports, Fujian Jiangxia University, Fuzhou, China; 4 Department of Sports Rehabilitation, Hunan University of Medicine, Huaihua, China; Harbin Institute of Technology, CHINA

## Abstract

The global manufacturing landscape is undergoing a profound shift, and the paradigm of service-led industrial evolution has taken on paramount importance. Service-oriented manufacturing has emerged as a pivotal conduit for the transformation and elevation of China’s sporting goods manufacturing sector. However, we acknowledge that the current state of service-oriented transformation in this sector is still in an embryonic phase. The nuanced interplay between the level of servitization and its consequent impact on corporate performance, along with the intricate forms of interference exerted by various factors in the process of servitization, are shrouded in ambiguity. This article, which is predicated upon the financial disclosures of China’s sporting goods manufacturing enterprises listed on the A-share and New Third Board markets, presents a rigorous investigation. Through the construction of an imbalanced panel dataset covering the period of 2007 to 2022, we embark on an empirical journey examining the intricacies of a moderated mediation model to ascertain the mechanisms underlying the influence of servitization on the performance of sporting goods manufacturing entities. Through this research, we unearth a compelling revelation: (1) Within the cohort of sampled enterprises, an elevation of servitization evokes a modest suppressive effect on corporate performance, thus resulting in the enigmatic "servitization paradox." (2) A total of 29.1% of the servitization impact on enterprise performance is achieved through marketing intensity; that is, there is a partial mediating effect of marketing intensity on the relationship between servitization and enterprise performance. (3) Market power play a negative regulatory role in this relationship, which drives the servitization paradox in sporting goods manufacturing but can also promote the positive impact of servitization on marketing intensity. In addition, with the expansion of market power, corporate servitization does not need to impact corporate performance through marketing activities. (4) R&D intensity negatively affects the relationship between marketing intensity and corporate performance, promotes the inhibitory effect of marketing intensity on corporate performance, and aggravates the inhibitory effect of servitization on corporate performance through marketing intensity.

## Introduction

The sports industry has undergone the fastest growth rate since entering the 2020s. According to the PR Newswire, the market value of the global sports industry is growing at a compounded annual growth rate of 8.1% per year and is projected to reach US $253.465 billion by 2024 [1]. As a part of China’s "Five Happy Industries", the sports industry totaled 2737.2 billion yuan in 2020, accounting for 2.7% of GDP, with an added value of 1073.5 billion yuan, and the economic contribution of the sports industry will be significantly enhanced in the future. The sporting goods manufacturing industry is an important part of the sports industry, and the total output and added value of the national sporting goods and related products manufacturing industry in 2020 was 1228.7 billion yuan and 314.4 billion yuan, accounting for 44.9% and 29.3%, respectively, of the total value of the sporting industry. However, in recent years, through the embedding of OEMs and international OEMs in the global value chain of China’s sporting goods manufacturing industry, the development, direction and revenue in this sector has been increasingly restricted, thus shrinking external demand and weakening the domestic demographic dividends that have resulted from the continued increase in labor costs, and causing sales revenue and profits to fall sharply; additionally, numerous well-known domestic sporting goods brands are facing a wave of store closures and a continued decline in the pressure that drives transformation and upgrading, but at the same time, the enormous domestic product service market size and capacity also provide a broad space for the extension of China’s sporting goods manufacturing industry chain and opportunities for technological upgrading and the transformational innovation of products. In a competitive service environment, service innovation is one of the strategic routes for company survival and growth enhancement This is because the practice of service innovation introduces new services and processes that cater to shifting preferences, tastes, and choices [2]. The policies introduced by the Chinese government at the appropriate times have provided support for the servitization of sporting goods manufacturing enterprises. In the *Made in China 2025* [3] announcement that was issued by the State Council in 2015, "actively developing service-oriented manufacturing and productive service industry" rose to the level of a strategic task, establishing the path of synergistic development of manufacturing and service and clarifying the development goal of transforming production-oriented manufacturing to service-oriented manufacturing (SOM). In 2019, the State Council issued the *Opinions on Promoting National Fitness and Sports Consumption and Promoting the High-quality Development of the Sports Industry* [4], which proposed the need to increase the share of the sports service industry and support the innovative development of the sporting goods manufacturing industry.

The concept of servitization, which was initially proposed by Levitt in 1972 [5] in the context of analyzing industrial substitution and competitive advantages in developing countries as compared to those in developed countries, posits that servitization is "the process by which companies transition from providing pure products to delivering a combination of products and services to create value." Servitization essentially represents the transformation of a company from purely providing products to solely delivering services [6]. In the manufacturing industry, while leading the transformation and upgrading of China’s manufacturing industry, servitization can also enhance the value of the enterprise’s products and promote the improvement of enterprise performance [7]. Combining sporting goods manufacturing with services enables the extension of the product value chain to both ends of the "smile curve" in SOM [8]. Simultaneously, this integration increases the added economic value of China’s sporting goods manufacturing industry, elevates the perceived quality of customer sports consumption, and extends the lifecycle of sporting products [9]. In recent years, an increasing number of China’s sporting goods manufacturing enterprises have initiated an expansion of the sports service industry. For example, through the development and enhancement of service business in the upstream R&D and design stage, ANTA Sports Products Ltd. a shift in its products from a focus on physical properties to an emphasis on both performance and customer experience; additionally, they upgraded themselves from a product manufacturer to a product manufacturing and service integrator and greatly enhanced the irreplaceability of its products by increasing the service functions attached to those products. 361 Degrees International Ltd., a firm that relies on modern internet technology and Baidu, Inc., to engage in cooperation, set up a "big data innovation laboratory", which relies on a big data network to upgrade the services of both the upstream and downstream links of the industry chain, such as R&D and sales. They provide complete value-added services and create a total solution involving both products and services to meet individual customer needs. However, as early as 2005, Gebauer, working in German machine tool industry research, found that machine tool manufacturers failed to obtain the expected profit from the addition of service and therefore presented the concept of the service paradox [10]. This phenomenon refers to the fact that some manufacturers may not be able to generate sufficient revenues from their servitization efforts despite making significant investments in their service business; i.e., an increase in the level of firm servitization does not improve firm performance and may even have a dampening effect. Neely [11] collected revenue data from 10028 companies in 25 countries and further confirmed the existence of the servitization paradox. Kastalli et al. [12] used a well-known Swedish company as an example and found that through the innovation of a business model, an enterprise can obtain a relative strategic advantage during the initial stage; however, with the expansion of its service business, the original resources of the enterprise become consumed, resulting in the enterprise sacrificing other businesses aspects to protect its service-oriented strategy.

At present, the promotional effect of servitization on enterprise performance in China’s sporting goods manufacturing industry is limited by theoretical discussion. Although studies have confirmed the existence of the servitization paradox in the manufacturing industry, whether this phenomenon also exists in China’s sporting goods manufacturing industry has not been determined. China’s sporting goods manufacturing enterprises are still in their initial stage of service-oriented transformation; the vast majority of small and medium-sized enterprises in the industry are still relatively weak in service-oriented awareness, the concept of service-oriented transformation is still immature, and the shortage of specialized personnel who can promote the high-quality development of enterprises through service-oriented transformation is highly challenged. Therefore, the following questions must be answered: (1) Can the implementation of the service-oriented transformation of Chinese sporting goods manufacturing enterprises truly improve enterprise performance? (2) Does the servitization paradox exist in Chinese sporting goods manufacturing enterprises? (3) In the process through which service-oriented transformation affects enterprise performance, do internal enterprise factors play moderating or mediating roles, and what are the respective effects of these factors?

### Literature review

#### Servitization, enterprise performance and their influencing factors

After the concept of servitization was proposed by Levitt [5], Vandermerwe and Rada [13] extended it to the manufacturing industry, arguing that servitization represents a shift in the focus of manufacturing enterprises from an operational mode of providing only tangible products or of providing tangible products and their after-sales services to the operation mode of providing product plus service packages. Later, this phenomenon gradually evolved to refer specifically to the transition from selling products and after-sales services to providing more advanced services in the form of integrated total solutions [14]; however, at present, servitization is still a new theoretical field [15]. The relationship between servitization and enterprise performance can be summarized into the following categories: (1) Positive facilitation theory: Most of the early scholars claim that there is a positive relationship between manufacturing servitization and enterprise performance [16], and subsequent studies have also verified this claim from the perspectives of service innovation [14], the evolution of servitization [17], and the transformation and upgrading of the manufacturing industry [18]. (2) Negative inhibition theory: In 2005, Gebauer et al. [19] first proposed the servitization paradox phenomenon. Neely [11] was the first to verify this claim through enterprise financial data and found that the rate of declared bankruptcy in manufacturing enterprises engaged in service-oriented strategic transformation is much greater than that of pure manufacturing enterprises. Benedettini et al. [20] further discussed the reasons for this phenomenon from the perspective of service breadth and enterprise survival. In China, Weng et al. [21] previously analyzed the mechanism underlying the negative impact of servitization transformation on enterprise performance from the perspective of financial and marketing differentiation. Xiao [22] further confirmed that the total number of service business types (breadths) involved in enterprises is negatively related to corporate profit margins and market value. (3) Complex relationships and noncorrelations: After the servitization paradox was first proposed, scholars further uncovered more complex relations, including the positive "U" type [23], inverted "U" type [24], saddle type [25] and no correlation [26]. Regarding the research on the factors influencing servitization, Benedettini et al. [27] summarized 76 factors that affect enterprise servitization from the perspective of enterprise business processes based on the four aspects of the enterprise market and product, technology, the production process and administrative management. The factors that affect the relationship between servitization and corporate performance are generally classified into two categories: corporate characteristics and environmental factors. Enterprise characteristics cover the technology, production and management of service-oriented products, such as the progress of service-oriented products and their technical combination ability with main products, the efficiency of service input and service output, and the construction of interactive networks between enterprises and customers [28]. The characteristics of environmental factors include the heterogeneity of the enterprise field, the competitive intensity of market growth, the development of product marketing promotion platforms, and the restriction of the regulatory environment [29, 30]. Generally, both domestic and international studies apply empirical models to explore the effect of manufacturing servitization performance or focus on the implementation path of manufacturing servitization for improving enterprise performance through theoretical and case analyses; however, a consensus on the effect of servitization on firm performance has yet to be reached, and the research on the mechanism of the effect of servitization on the performance of China’s manufacturing industry, particularly for sporting goods manufacturing enterprises, has also failed to reach a consensus.

### The relationship between marketing intensity and firm performance

Marketing intensity refers to the cost of a firm’s investment in marketing activities, and most of the current research in the academic community has concluded that marketing intensity contributes to improving firm performance. Pitelis [31] analyzed the financial data of several listed companies in the United Kingdom and found that there was a very significant improvement in operating income after companies increased their investment in marketing expenses. Srinivasan [32] analyzed the financial data of 3,804 publicly traded companies in the U.S. from 1969 to 2007 and found that investment in advertising and marketing expenses positively and significantly boosted corporate profits. In a study of Chinese enterprises, Niu et al. [33], based on relevant public data of Chinese industrial enterprises, found that the marketing intensity of Chinese industrial enterprises has a significant positive effect on both the growth rate of business revenue and the growth rate of profit. Li et al. [34], using data from dairy companies, found that marketing investment can promote the improvement of corporate performance. Zhou [35] utilized the data of manufacturing enterprises, embedded information on financial leverage in the influence of marketing input on enterprise performance, and found that marketing input can offset the negative influence of financial leverage on enterprise performance to a certain extent. However, at the same time, the impact of marketing inputs on firm performance also has a double-edged sword effect, which exerts both a crowding-out effect and a facilitating effect. If enterprises can capitalize their marketing investment within the scope allowed by accounting standards and spread the marketing investment to their future long-term perspective, then the growth of enterprise performance becomes inhibited [36]. Using listed companies in China’s pharmaceutical industry as an example, Luo [37] found that although sales expense expenditures are significantly positively correlated with corporate performance, the marketing profit margin decreases after the operating profit margin reaches a specific percentage of marketing expenditure. Abbott’s [38] study showed that firms’ marketing investment is positively related to the sales revenue of their products in the short term, but advertising investment has a significant positive effect on shareholder value only when the firm conducts a new product launch. Hirschey [39] considers the firm size factor in an empirical test and finds that marketing advertisements have a sustained positive effect on the market value of large-scale firms only. Overall, in studies that address the relationship between marketing intensity and firm performance, most findings show a positive effect, while a small number of studies suggest that the amount of marketing allocation, firm size and different characteristics of the life cycle can exert a negative impact on firm profits, given a certain level of overall firm resources.

### The servitization transformation of sporting goods in China

The related research in China has focused mainly on cooperative R&D alliances in industry and university research in sporting goods manufacturing enterprises as well as the regional brand effect formed by industrial agglomeration, service production cooperation, etc. In recent years, with the promotion of the *Made in China 2025* strategy, numerous scholars have discussed the servitization development status in China’s sporting goods manufacturing industry from various perspectives and have proposed various implementation strategies. Li et al. [7, 8] analyzed the innovation path of the basic elements of the business model of China’s sporting goods industry with the help of value chain theory and discussed the service innovation model of Anta Enterprise in detail. Zhao et al. [40] considered the constraints of China’s sporting goods manufacturing industry, such as a lack of funds, weak independent research and development capabilities, a lack of high-end compound talent, and an imperfect industrial ecology, and proposed strategies such as the promotion of scientific and technological innovation, the improvement of talent engineering, and the optimization of the business environment. Duan et al. [9] also analyzed the role of servitization in the transformation and upgrading of China’s sporting goods industry and concluded that the servitization transformation can help to build a multilevel sporting goods service supply system, enhance the economic value added to China’s sporting goods manufacturing industry, improve customer perceptions of sports consumption, and prolong the life cycle of sports products. On this basis, Liu [41] further proposed countermeasures to these issues, such as improving the SOM policy system, promoting the demonstration of typical SOM models, strengthening basic capacity building, innovating internet business and service models, and strengthening the introduction and training system of talent. Pan et al. [42] expounded on the promotion of China’s sporting goods manufacturing industry from three dimensions: productivity innovation, industrial chain extension and product value promotion.

Throughout the relevant research on the servitization of China’s sporting goods manufacturing industry, two issues require further explanation: (1) The current empirical analysis of the impact of servitization on enterprise performance is relatively plentiful, but that analysis regarding the sporting goods manufacturing industry is relatively limited. Most of the existing studies are based on logical discourse or apply case study paradigms to explore the development mechanism, power structure and realization path analysis of the service-oriented transformation of the sporting goods manufacturing industry from macro and meso perspectives [9, 41–43], as well as applying case studies of specific regions and representative enterprises [7, 8, 40]; however, there is a lack of exploration of the factors affecting the service-oriented development of sporting goods manufacturing enterprises from the micro perspective. (2) Servitization can promote the extension of China’s sporting goods manufacturing enterprises both upstream and downstream of the value chain and enhance the status of China’s manufacturing industry in the labor division of the global value chain. However, because of the difficulties in screening the servitization business of Chinese sporting goods manufacturing enterprises and the constraints posed by data availability, there is a dearth of relevant research on the empirical analysis of the impact of servitization on the performance of sporting goods manufacturing enterprises at the micro level, as well that on the impact and regulatory mechanism of the intrinsic (extrinsic) role of the internal (external) paths and mechanisms underlying that impact. Closing this research gap is a high-value goal. Therefore, the main contribution of this paper is to clarify these issues on the basis of previous studies by fully mining the financial data of Chinese sporting goods manufacturing enterprises; accurately measuring the level of enterprise servitization and related intermediary and regulating variables; and constructing relevant empirical models, which not only assists in clarifying the current servitization transformation of China’s sporting goods manufacturing industry but also provide realistic references for the successful implementation and smooth operation of the servitization strategy in other relevant enterprises.

## Theoretical analysis and research hypotheses

### Servitization and enterprise performance in sporting goods manufacturing enterprises

With the gradual expansion of the research on servitization and the further deepening of related research, relevant studies have shown that the service innovation activities of manufacturing enterprises lead to a decrease in overall profit levels, and servitization strategy plays a role in the promotion of enterprise performance only in specific circumstances [26]. Whether an enterprise services proves complementary to the original products determines the positive impact of the enterprise servitization on the business [44]. An enterprise’s inability to address the problems of servitization investment and internal resource allocation leads to a substantial decline in corporate profitability and thus results in the servitization paradox. Although relevant studies have fully explained the positive role of servitization in enhancing the transformation and upgrading of China’s sporting goods manufacturing industry [40–42], they remain limited to theoretical analysis. Under the fourth global industrial transfer, external demand has shrunk, the land, labor and production costs of Chinese sporting goods enterprises have risen, and both sales revenue and profit have fallen sharply [45]. This has also pushed the production mode of Chinese sporting goods manufacturers to gradually transform from an original equipment manufacturer (OEM) to an original brand manufacturer (OBM). To enhance market influence, major manufacturers have been increasing their investment in research and development (R&D) in an effort to differentiate themselves in the market through serious product homogenization and the expansion of their market share. However, the correlation between servitization and product technology innovation cannot be explained by complementarity alone; due to resource competition, product innovation in many contexts consumes corporate resources and can exert a negative impact on servitization [46]. Excessive investment in service-oriented businesses can cause a "squeeze-out effect" on product innovation and R&D, and the failure to produce products that meet the demands of the market and are welcomed by consumers can negatively affect enterprise performance. For some small and medium-sized sports manufacturing enterprises who take production and processing as their main business, the senior management team lacks sufficient knowledge and understanding of the service-oriented strategy and lacks enthusiasm for the development of service-oriented business [47].

In addition, the structure of China’s sporting goods manufacturing market is still in a transition growth period, the number of listed companies remains at an absolute disadvantage compared to that of other industries, and most companies have been listed for less than seven years and are still in the growth period of the enterprise life cycle. Of the 37 listed Chinese sporting goods enterprises, the average enterprise service business accounted for only 2.636% of enterprise sales during the 2010–2022 period, the business volume was small, and the development of service-oriented transformation was still in its early exploration stage. In addition, the shift from selling finished products to providing corresponding services requires not only technical transformation but also the cognitive transformation of business managers and the diversification of the capabilities of business employees [48]. In the process of servitization, factors such as organizational inertia [49], corporate culture [50], and cognitive barriers [51] can have negative impacts on firm performance, which poses challenges to Chinese listed sporting goods firms in the process of servitization transformation. Thus, the following hypothesis is proposed:

H1: A "servitization paradox" phenomenon occurs in the relationship between servitization and enterprise performance in sporting goods manufacturing enterprises.

### Mediating effect of the marketing intensity of sporting goods manufacturing enterprises

Marketing intensity refers to the degree to which manufacturing enterprises invest financial resources in their marketing behavior or market development. To a certain extent, the marketing resources and investment of an enterprise reflect its level of support for product strategy [52]. Day [53] divided marketing capabilities into three dimensions: internal-external capabilities, such as effective cost control, information storage and logistics support, which are all conducive to gaining a competitive advantage; external capabilities, such as customer and competitor research and the establishment of relationships with customers and suppliers; and leapfrogging capabilities enabled through the integration of the above two capabilities for the development of efficient strategic execution capabilities.

Marketing intensity can be seen through the efforts that a company is willing to expend to promote its products or services in the marketplace, and the term encompasses all the activities of a company that facilitate communication with the outside market [52]. Marketing activities enhance the ability of enterprises to release signals to the market; thus, for the servitization business of sporting goods manufacturers, a strong marketing ability increases market acceptance of the ancillary services they developed and reduces the risks associated with servitization. Moreover, through marketing, companies can "incubate" potential customers, thus expanding the market scope and acceptance of their products and services [54]. In addition, through marketing activities, companies can collect consumer feedback information in a timely manner to better understand the current customer needs and market trends, which in turn assists them in adjusting their business strategies and improving their brand value and level of market competitiveness [55]. When a company launches a new product (new goods or new services), it is difficult for consumers to realize its value prior to experiencing it. Therefore, marketing activities are necessary, as they can help consumers identify needs and can help companies develop new markets [56]. The marketing activities of sporting goods manufacturing companies can achieve the differentiated positioning of main products and service-oriented products and can win brand equity and service competitive advantage by conveying positive messages to investors and through effective and rapid process and activity configuration. Thus, the following hypothesis is proposed:

H2: Marketing intensity plays a mediating role in the relationship between servitization and enterprise performance for sporting goods manufacturing enterprises.

### Regulating effect of market power and the r&d intensity of sporting goods manufacturing enterprises

Market power is also an important means for enterprises in the same industry to gain a competitive advantage, and it comprehensively reflects enterprise capacities for pricing and market monopoly. Wise et al. [57] determined that market power served as an important strategic tool in the early days of manufacturing servitization. In the context of manufacturing servitization strategy, market power plays a key role in the relationship between manufacturing enterprise service transformation and enterprise performance. From the perspective of service input and output, manufacturing enterprises can build market barriers, expand market power, build long-term and stable marketing mechanisms, and win competitive advantage by leveraging large capital investment [58]. For sports manufacturing enterprises of different sizes and life cycles, the choice of service-oriented business differential marketing or indifferentiable marketing strategy depends on the factors of product design capacity, product quality, brand reputation and management quality. These capacity differences also reflect the market position of enterprises. The leading enterprises in the industry have more stable customer bases, their marketing channels are more stable, and their proportion of marketing investment is relatively lower. In the sports manufacturing market segment, internal enterprises construct "strong entry barriers" to external enterprises to maintain stable marketing channels, ensure the profits of internal enterprises in the market, and maintain their price monopoly ability. As market power grows, the monopoly position of the servitization business intensifies at the industry level, which is not conducive to the horizontal comparison of customers or to healthy competition in the servitization business that serves to improve service level and enterprise performance. Thus, the following hypothesis is proposed:

H3a: Market power negatively regulate the relationship between servitization level and corporate performance; that is, market power promote the "servitization paradox."
H3b: Market power positively regulate the relationship between servitization level and marketing intensity.
H3c: Enterprises with greater market power have weaker levels of dependence on servitization for improving corporate performance through marketing intensity.

R&D investment refers to the innovative behavior and resource investment taken by manufacturing enterprises to stand out from their competition [59]. The manufacturing servitization process reflects the innovative thinking of enterprises, and enterprises need to creatively transition from selling products to selling services [60]. The R&D of new products can affect consumer expectations regarding product benefits and subsequently affect the performance of enterprises. R&D innovation plays a mediating role between marketing activities and enterprise performance, and innovation activities can promote the development of marketing activities and, in turn, bring economic benefits to enterprises [61]. However, some studies have shown that the R&D investment of listed sporting goods enterprises has no significant effect on corporate performance [62]. At present, most sporting goods manufacturing enterprises are in the middle reaches of the industrial chain, have low added value and profits and low requirements for technology, and are part of the production-oriented manufacturing mode [7]. The input of manpower, material and financial resources in R&D and marketing activities exerts great pressure on the production costs of the enterprise, which can constrain the improvement of enterprise financial performance. Thus, the following hypotheses are proposed:

H4a: R&D intensity negatively modifies the relationship between marketing intensity and enterprise performance and enhances the burden of marketing intensity on enterprises.
H4b: The improving effect of marketing intensity on corporate performance is constrained in enterprises with high levels of R&D intensity.

**Fig 1:**
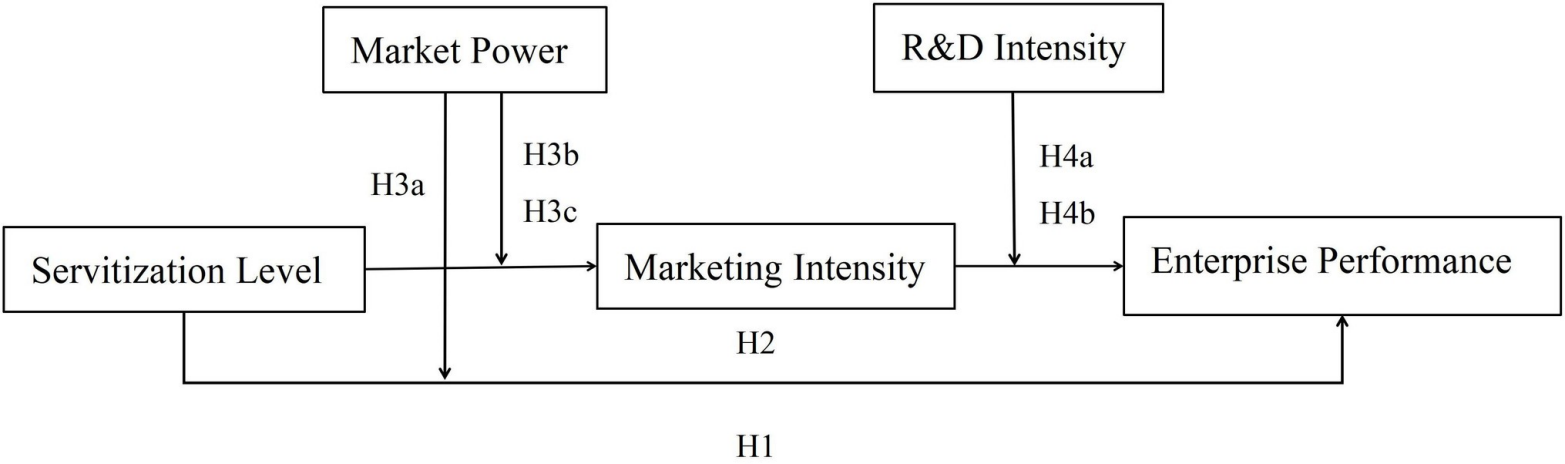
Theoretical framework.

Based on the above analysis, the theoretical research framework is shown in Fig 1.

## Research design

### Sample selection and data sources

The shares of sporting goods manufacturing enterprises listed on the Shanghai Stock Exchange, Shenzhen Stock Exchange and New OTC Market are selected as sample frames, and the selection criteria are as follows: (11) the selected listed sports enterprises must comply with the relevant provisions of the national sports industry statistical classification and carry out service business during the year of listing; (2) the business scope of the listed enterprises must include sporting goods manufacturing, have its own brand, and sporting goods manufacturing must account for more than 50% of the enterprise’s total revenue; (3) there are no major financial mistakes in the enterprise’s annual report; (4) the enterprises are excluded from the year samples of ST, SST, S*ST, * ST or PT; and (5) selected enterprises must have continuous sample data for at least 3 years. The financial indicators are taken from the Guotai’an database, the Juchao Information Network and annual enterprise report data. The basic information of the 37 sporting goods manufacturing listed enterprises, with a total of 286 sample data points, is shown in Table 1.

**Table 1:** Basic information of the study sample.

Security Code	Enterprise Dame	Data Period	Security Code	Enterprise Dame	Data Period	Security Code	Enterprise Dame	Data Period
002780	Sanfo Outdoor	2015–2022	603908	Comefly	2017–2022	833649	Beaume Outdoor	2015–2022
600679	Shanghai Phoenix	2010–2022	002899	Impulse	2017–2022	836210	Sumar Marine	2017–2022
300526	China Dive	2016–2021	300651	Jinling Sports	2017–2022	833151	Tongfang Health	2015–2022
002105	HL Corp	2007–2022	002870	Senssun	2017–2022	870749	Jianhua Zhongxing	2017–2022
603558	Jasan Group	2015–2022	603555	Guirenniao	2014–2018	833429	Competitor	2015–2022
002395	Double Elephant	2010–2022	831326	Sanlida	2014–2022	833603	Aosen Garment	2015–2022
002486	Challenge	2010–2022	873009	Sifang Swimming	2018–2022	832875	Fushide	2015–2022
300005	Toread	2010–2022	838464	Carving Ski	2016–2022	830877	Kanglai Sports	2014–2022
603129	Chunfeng Power	2017–2022	430759	Cronus	2014–2017	871594	Cnsg Holdings	2017–2022
002832	Biemlfdlkk	2016–2022	837226	Lianchuang Artificial Lawn	2017–2022	837720	Youli Sports	2016–2022
002489	Zhejiang Yongqiang	2010–2022	834261	Inov	2016–2022	871721	Source One Outdoor	2017–2022
835558	Yihong Yacht	2016–2022	430574	Singolym Technology	2014–2019	839387	Yodo Group	2016–2022
872531	Teloon Tennis	2017–2019						

Note: Guirenniao was *ST in 2019–2020 and ST in 2021; China Dive was *ST in 2022; Cronus was suspended after 2018; Teloon Tennis and Singolym Technology were suspended in 2020.

### Variable selection and data description

#### Enterprise performance *(ROA)*

An enterprise or other socioeconomic organization is regarded as a legal person for the purpose of profit; return on assets (*ROA*) directly represents the financial situation of the enterprise and can more truly reflect profitability [63]. Moreover, referring to the research of Zhu et al. [64], *ROA* is selected as the performance index for sporting goods manufacturing enterprises in the benchmark regression model and is calculated by dividing the current year’s net profit before tax by total assets.

#### Servitization level (*SER*)

With reference to the existing relevant research methods used to measure the servitization level of manufacturing enterprises, the proportion of service-oriented income in total business income is used to measure the servitization level of sporting goods manufacturing listed enterprises [65, 66]. Several scholars have conducted special research on the accuracy of this alternative method, and the results show that it is practical and reliable and also serves an objective micro index for the measurement of enterprise service income [67]. In addition, the term "other business income" may include income from the sale of materials and income from the transfer of intangible assets. Therefore, the value after excluding these items is regarded as the revenue of the service-oriented business, and the proportion of this value to the enterprise’s operating income is used to represent the level of corporate servitization. For some enterprises that do not publish the details of their other business income, this factor is used as the service income indicator.

#### Mediating and regulatory variables

*Marketing intensity (MAR)*. Marketing intensity is generally measured by marketing investment [68]. The capital investment in marketing is usually managed in the form of sales expenses, which can reflect the investment of enterprises in market expansion, product promotion, etc. Drawing on the measurement method of Zhou [35], the current marketing investment is measured by the ratio of sales expenses to total operating income in an enterprise’s financial statements.

*R&D intensity (RD)*. Product innovation represents the investment of enterprises in product innovation behavior, and R&D intensity has always been considered one of the most important factors affecting the product and process innovation activities of manufacturing enterprises. Following the practice of Josephson et al. [69], R&D intensity is measured by the ratio of R&D investment to total revenue.

*Market power (MKT)*. The Lerner index is currently recognized as the main indicator of market power, and its calculation method is as follows: MKP = (P—MC)/P, where MKP, P and MC represent the Lerner index, product price and marginal cost, respectively. Limited to the acquisition of product price and marginal cost data, this work draws on the operation method of Kale [70] to improve the Lerner index, replacing product price and marginal cost accounting market power with main business income and main business cost.

#### Control variables

The factors that affect enterprise performance are diverse. According to relevant studies, enterprise size, enterprise age, enterprise capital intensity and the enterprise asset-liability ratio are selected as control variables. The detailed measurement of each variable is shown in Table 2. The financial index data of the sample companies over past years were obtained from the database of Cathay Pacific (https://www.gtarsc.com/) and the Juchao Information Network (http://www.cninfo.com.cn/new/index).

**Table 2:** Variable names and measures.

Variables		Symbols	Definition and Measurement
Dependent variable	Enterprise Performance	*ROA*	Net Profit/Total Assets*100
Core independent variable	Servitization Level	*SER*	Service-Oriented Business Revenue/Enterprise Sales *100
Mediating variables	Marketing Intensity	*MAR*	Sales Expenses/Total Revenue*100
Adjusting variables	Market Power	*MKT*	(Main Operating Income-Main Operating Cost)/Main Business Income
	R&D Intensity	*RD*	R & D Investment/Total Revenue*100
Control variable	Enterprise Scale	Ln*SIZE*	Natural Logarithm of Total Assets of An Enterprise
	Enterprise Age	Ln*AGE*	Natural Logarithm of The Data Year Minus The Year of Establishment + 1)
	Capital Intensity	Ln*KLR*	Natural Logarithm of (Net Fixed Assets/Number of Employees)
	Asset-Liability Ratio	*DEDT*	Total Liabilities/Total Assets

### Model building

According to the previous analysis of the correlation and transmission mechanism between servitization level and the performance of sporting goods manufacturing enterprises, the time-individual two-way fixed effect model is introduced through the Hausman test to solve the endogeneity problems caused by unobservable factors to some extent.

(1) Direct effect and mediating effect. To test the influence of the corporate servitization level on corporate performance, as well as the mediating role played by marketing intensity, the following model is constructed by drawing on the mediating effect testing method proposed by Wen et al. [71]:

$$ROA_{it}=\beta_{0}+\beta_{1}SER_{it}+\beta_{2}controls+\mu_{t}+\lambda_{i}+\varepsilon_{it}$$ (1)

$$MAR_{it}=\alpha_{0}+\alpha_{1}SER_{it}+\alpha_{2}controls+\mu_{t}+\lambda_{i}+\varepsilon_{it}$$ (2)

$$ROA_{it}=\gamma_{0}+\gamma_{1}SER_{it}+\gamma_{2}MAR_{it+}\gamma_{3}controls+\mu_{t}+\lambda_{i}+\varepsilon_{it}$$ (3)

where *i* represents the enterprise, *t* represents the year, *ROA* represents enterprise performance, *SER* represents the enterprise servitization level, *MAR* represents the marketing intensity, and *controls* represent the control variables. Moreover, the annual fixed effect (*μ*_*t*_) and provincial fixed effect (*λ*_*i*_) are controlled in the model, and *ε*_*it*_ is a random disturbance term, and the same applies below.

(2) Moderating effect. First, the moderating effect of market power on servitization and firm performance is tested, and Eq (4) is obtained if the interaction between servitization and market power is introduced on the basis of Eq (1), Eq (5) is constructed on the basis of Eq (2), and Eqs (6) and (7) are used to test the moderating effect of R&D intensity on marketing intensity and enterprise performance.

$$ROA_{it}=b_{0}+b_{1}SER_{it}+b_{2}MKT_{it}+b_{3}SER_{it}*MKT_{it}+b_{4}controls+\mu_{t}+\lambda_{i}+\varepsilon_{it}$$ (4)

$$MAR_{it}=c_{0}+c_{1}SER_{it}+c_{2}MKT_{it}+c_{3}SER_{it}*MKT_{it}+c_{4}controls+\mu_{t}+\lambda_{i}+\varepsilon_{it}$$ (5)

$$ROA_{it}=d_{0}+d_{1}MAR_{it}+d_{4}controls+\mu_{t}+\lambda_{i}+\varepsilon_{it}$$ (6)

$$ROA_{it}=e_{0}+e_{1}MAR_{it}+e_{2}RD_{it}+e_{3}MAR_{it}*RD_{it}+e_{4}controls+\mu_{t}+\lambda_{i}+\varepsilon_{it}$$ (7)

(3) Moderated effects. To further test the mediating effect with moderation, following Wen and Ye [72], Eqs (8) and (9) are constructed to test whether market power and R&D intensity can modulate the relationship between servitization and firm performance through marketing intensity.

$$ROA_{it}=f_{0}+f_{1}SER_{it}+f_{2}MKT_{it}+f_{3}SER_{it}*MKT_{it}+f_{4}MAR_{it+}f_{5}controls+\mu_{t}+\lambda_{i}+\varepsilon_{it}$$ (8)

$$ROA_{it}=g_{0}+g_{1}MAR_{it}+g_{2}RD_{it}+g_{3}MAR_{it}*RD_{it}+g_{4}SER_{it+}g_{5}controls+\mu_{t}+\lambda_{i}+\varepsilon_{it}$$ (9)

*MKT*_*it*_ and *RD*_*it*_ represent market power and R&D intensity, respectively. To reduce nonessential collinearity between independent variables and regulatory variables, each interaction term is centralized. The level of servitization after SER*MKT-centered processing is the interaction item of market power and the interaction item of marketing intensity and R&D intensity after MAR*RD-centered treatment; the other variables are the same as those used above.

## Empirical results

### Descriptive statistics and correlation analysis

To avoid the influence of data outliers, all variable data are tailed with a 1% winsorization rate. Table 3 presents the descriptive statistics and correlation analysis of all the variable samples involved in the research hypothesis. In the correlation analysis, the Pearson coefficient and Spearman coefficient are used to test each variable. The absolute value of the correlation coefficient of the variables used in this paper is not more than 0.70, and the VIF value ranges from 1.14 to 1.55; that is, there is no problem of multicollinearity, which ensures the accuracy of the latter regression results.

**Table 3:** Results of descriptive statistics and correlation analysis.

	*ROA*	*SER*	*MAR*	*MKT*	*RD*	Ln*SIZE*	Ln*AGE*	Ln*KLR*	*DEBT*
*ROA*	1	-0.090	-0.010	0.294***	-0.018	-0.007	-0.275***	-0.158**	-0.130**
*SER*	-0.161**	1	0.025	-0.061	-0.011	0.080	0.152**	0.267***	-0.054
*MAR*	-0.142**	0.114*	1	0.671***	0.358***	-0.072	-0.098*	-0.107*	-0.155**
*MKT*	0.213***	-0.042	0.465***	1	0.470***	-0.250***	-0.123**	-0.087	-0.353***
*RD*	-0.187**	-0.071	0.346***	0.285***	1	-0.341***	-0.099*	0.024	-0.376***
Ln*SIZE*	0.045	0.008	-0.026	-0.172**	-0.324***	1	0.303***	0.295***	-0.098
Ln*AGE*	-0.108*	0.125**	-0.025	-0.047	-0.069	0.306***	1	-0.068	-0.014
Ln*KLR*	-0.110*	0.186**	-0.092	-0.114*	-0.040	0.320***	-0.081	1	-0.072
*DEBT*	-0.148**	0.035	-0.314***	-0.322***	-0.180**	-0.123**	-0.041	-0.075	1
Mean	3.679	2.727	10.292	0.259	3.589	20.070	2.801	11.521	0.408
SD	8.10	4.881	9.737	0.201	1.991	1.520	0.346	1.246	0.181
VIF	—	1.14	1.52	1.42	1.36	1.55	1.20	1.28	1.23

Note: (1) The lower left corner shows the Pearson correlation coefficient test result, and the upper right corner shows the Spearman correlation coefficient test result; (2) *, **, and **** indicate significance at the 10%, 5%, and 1%, levels, respectively (double-tailed test).

### Hypothesis test

#### Direct effect and mediating effect tests

Columns (1)-(3) in Table 4 correspond to Models (1)-(3), respectively. Obviously, there is a significant negative relationship among the servitization level of sporting goods manufacturers, the performance of enterprises and the total effect, *β*_*1*_ = -0.4019 (*p*<0.05); that is, the "servitization paradox" occurs, and H1 is verified. Xu et al. [73] and Eggert et al. [26] confirmed this phenomenon among manufacturing enterprises in China and Germany, and we confirm it here in the Chinese sporting goods manufacturing industry. Second, the mediating effect of enterprise marketing intensity was tested, and the level of enterprise servitization had a very significant positive effect on marketing intensity (*α*_*1*_ = 0.2862, *p*<0.001). As Lu [56] noted, when a company launches a new product (new goods or new services), marketing activities can help consumers perceive the value of the product through experience. This is also in line with the claim that "product marketing has a broader space because of service-oriented transformation" [74]. After the addition of the marketing intensity variable, the level of servitization also had a significant negative impact on firm performance, with a direct effect of *γ*_*1*_ = -0.2951 (*p*<0.1); however, this change was weaker than that of the total effect. Moreover, marketing intensity had a significant negative impact on enterprise performance (*γ*_*2*_ = -0.3731, *p*<0.01), indicating that marketing intensity played a partial mediating role in the relationship between servitization level and enterprise performance, with a mediating effect value of -0.1168 and a mediating effect accounting for 29.1%. This finding supports the research on the complex relationship between marketing intensity and firm performance [38, 75], and also serves to remind business operators that if marketing intensity serves only to strengthen the product center rather than to support the firm’s servitization strategy, the market turbulence caused by the firm’s servitization strategy can become increasingly serious. Thus, H2 is verified.

**Table 4:** Hypothesis testing results.

	(1)	(2)	(3)	(4)	(5)	(6)	(7)	(8)	(9)
	*ROA*	*MAR*	*ROA*	*ROA*	*MAR*	*ROA*	*ROA*	*ROA*	*ROA*
*SER*	-0.4019**	0.2862**	-0.2851*	-0.0055*	0.0485**			-0.0147	-0.2408
	(0.1598)	(0.1313)	(0.1541)	(0.2045)	(0.1700)			(0.1922)	(0.1514)
*MAR*			-0.3731***			-0.3943***	0.1548	-0.4180***	0.1421
			(0.0771)			(0.0767)	(0.1619)	(0.0754)	(0.1615)
*MKT*				20.3020***	1.6647*			20.9979***	
				(4.1598)	(3.4582)			(3.9112)	
SER*MKT				-1.7396**	1.8764***			-0.9552	
				(0.6871)	-0.0485			(0.6610)	
*RD*							0.4630		0.3582
							(0.4977)		(0.5004)
MAR*RD							-0.0910***		-0.0853***
							(0.0254)		(0.0256)
Ln*SIZE*	5.4809***	-1.6263	4.8740***	5.2350***	-1.8211	5.3507***	3.9830**	4.4737***	(0.1615)
	(1.7237)	(1.4159)	(1.6492)	(1.6479)	(1.3699)	(1.6399)	(1.6298)	(1.5547)	3.6683**
Ln*AGE*	-12.1009*	2.1471	-11.2998	-11.4989	3.4533	-11.5337	-11.4242	-10.0553	(1.6363)
	(8.6715)	(7.1229)	(8.2741)	(8.2910)	(6.8925)	(8.3217)	(8.0960)	(7.7959)	-11.3552
Ln*KLR*	-1.3121	1.1006	-0.9015	-1.5494*	1.0667	-1.2549	-1.9286**	-1.1034	(8.0687)
	(0.8289)	(0.6809)	(0.7954)	(0.7933)	(0.6595)	(0.7782)	(0.7731)	(0.7498)	-1.6185**
*DEBT*	-14.7452***	0.3867	-14.6010***	-11.9405***	2.0389	-14.5062***	-13.2961***	-11.0881***	(0.7948)
	(4.3906)	(3.6065)	(4.1887)	(4.2511)	(0.036)	5.3507***	(4.0967)	(3.9980)	-13.4076***
Time/individual control	Yes	Yes	Yes	Yes	Yes	Yes	Yes	Yes	Yes
_cons	-55.7970*	25.3693	-46.3311	-55.8854	25.2737	-51.7830	-22.1733	-45.3198	-18.7490
	(40.3887)	(33.1760)	(38.5796)	(38.5763)	(32.0695)	(38.7001)	(38.2372)	(36.3024)	(38.1689)
N	286	286	286	286	286	286	286	286	286
R^2^	0.2145	0.1366	0.2792	0.3016	0.1918	0.2675	0.3173	0.3675	0.3249

Note: *, **, and **** indicate significance at the levels of 10%, 5%, and 1%, respectively (double-tailed test).

#### Moderating effect test

Columns (4)-(9) in Table 4 correspond to Models (4)-(9), respectively. According to Column (4), the interaction between the servitization level and market power has a significant negative effect on enterprise performance (*b*_*3*_ = -1.7396, *p<*0.05); *β*_*1*_ and *b*_*3*_ are the same in number; that is, market power promote the servitization paradox in sporting goods manufacturers, and thus H3a is verified. According to Column (5), the interaction between servitization level and market power has a significant positive effect on marketing intensity (*c*_*3*_ = 1.8764, *p<*0.01). Combined with the results of Column (2), these results show that the greater the market power of the enterprise is, the more that the servitization level can improve the marketing intensity of the enterprise, and thus H3b is verified.

The results of this study also further extend the research on the relationship between market power and servitization [57, 58]. In columns (6) and (7), marketing intensity and R&D intensity can be seen to be significantly negative (*e*_*3*_ = -0.091, *p<*0.01); that is, R&D intensity reinforces the inhibitory effect of marketing intensity on firm performance because it crowds out the firm’s capital and human resources. There is a significant mutual crowding out effect between innovation intensity and marketing intensity, which is also consistent with the results found by Guo [76] for Chinese A-share high-tech listed companies. Thus, H4a is verified.

#### Mediating effects and moderation testing

According to Eq (8), the interaction between servitization level and market power is negative but not significant, indicating that the expansion of market power makes corporate servitization unnecessary for the effect on corporate performance of marketing activities, and thus H3c is not supported. According to Model (9), after adding the multiplication term of marketing intensity and R&D intensity, *g*_*3*_ = -0.085 and *p*<0.01; further, *γ*_*1*_ and *g*_*3*_ are both negative, indicating that R&D intensity intensifies the inhibitory effect of servitization on firm performance through marketing intensity and thus H4b is supported.

### Robustness test

To alleviate the possible heteroscedasticity and correlation problems in the original model and solve the endogeneity problem to a certain extent, the regression was conducted using the "xtscc" command following the method of Yu et al. [77]. Second, with reference to Chen’s [78] research, the operating income growth rate (*OIGR*) was selected as a proxy variable for firm performance for robustness testing. Table 5 reports the results of the mediating effect of the two test methods, and Table 6 reports the results of the main steps of the moderating effect and the mediating effects with moderation of the two methods, which shows that the direction and significance of the regression coefficients of the main variables are basically consistent with those of the benchmark regression.

**Table 5:** Robustness tests for mediating effect.

Variables	Alternative Estimation Methods			Alternative Explained Variables		
	(1)	(2)	(3)	(4)	(5)	(6)
	*ROA*	*MAR*	*ROA*	*OIGR*	*MAR*	*OIGR*
*SER*	-0.4019***	0.2862**	-0.2851**	-0.0216***	0.2862**	-0.0149*
	(0.1153)	(0.1139)	(0.1034)	(0.0083)	(0.1313)	(0.0078)
*MAR*			-0.3731***			-0.0232***
			(0.0795)			(0.0039)
Control variable	Yes	Yes	Yes	Yes	Yes	Yes
Time/individual control	Yes	Yes	Yes	Yes	Yes	Yes
_cons	-55.7970	25.3693	-46.3311	0.4246	25.3693	1.0132
	(41.5444)	(31.5044)	(40.2087)	(2.0879)	(33.1760)	(1.9477)
N	286	286	286	286	286	286
R^2^	-	-	-	0.1226	0.1166	0.2419

Note: *, **, and **** indicate significance at the levels of 10%, 5%, and 1%, respectively (double-tailed test).

**Table 6:** Robustness tests for mediating effects with moderation.

Variables	Alternative Estimation Methods					Alternative Explained Variables				
	(1)	(2)	(3)	(4)	(5)	(1)	(2)	(3)	(4)	(5)
	*ROA*	*MAR*	*ROA*	*ROA*	*ROA*	*OIGR*	*MAR*	*OIGR*	*OIGR*	*OIGR*
*SER*	-0.4021***	0.2905***		-0.0147	-0.2408**	-0.0138**	0.0485*		-0.0150	-0.0159**
	(0.1774)	(0.1271)		(0.1677)	(0.0995)	(0.0111)	(0.1700)		(0.0103)	(0.0079)
*MAR*			-0.1549*	-0.4180***	0.1421			-0.0225***	-0.0248***	-0.0233***
			(0.1179)	(0.0653)	(0.1209)			(0.0084)	(0.0040)	(0.0084)
*MKT*	20.3020***	1.6647		20.9979***		0.2700	1.6647		0.3112*	
	(3.6784)	(6.0913)		(4.3822)		(0.2254)	(3.4582)		(0.2090)	
SER*MKT	-1.7396***	1.8764***		-0.9552*		-0.0360*	1.8764***		0.0105	
	(0.5453)	(0.3001)		(0.4809)		(0.0372)	(0.5712)		(0.0353)	
*RD*			0.4630		0.3582			-0.0284		-0.0353
			(0.4993)		(0.5304)			(0.0259)		(0.0260)
MAR*RD			-0.0910***		-0.0853***			-0.0001		-0.0003*
			(0.0186)		(0.0201)			(0.0013)		(0.0013)
Control variable		Yes	Yes	Yes	Yes		Yes	Yes	Yes	Yes
Time/individual control		Yes	Yes	Yes	Yes		Yes	Yes	Yes	Yes
_cons	-55.8854	25.2737	-22.1733	-45.3198	-18.7490	-0.4240	25.2737	-1.0554	1.0496	1.2813
	(36.9985)	(26.3297)	(40.2803)	(32.9977)	(39.5228)	(2.0898)	(32.0695)	(1.9900)	(1.9402)	(1.9796)
N	286	286	286	286	286	286	286	286	286	286
R^2^	-	-	-	-	-	0.1288	0.1818	0.2369	0.2545	0.2506

Note: *, **, and **** indicate significance at the levels of 10%, 5%, and 1%, respectively (double-tailed test).

## Conclusions and recommendations

Based on the financial data of China’s sporting goods manufacturing enterprises listed on the Shanghai and Shenzhen A shares and the New OTC Market during the period from 2007 to 2021, the effect mechanism of servitization on the performance of sporting goods manufacturing enterprises is verified through the construction of a moderating mediating model. The study reveals that (1) servitization inhibits the improvement of the performance of sporting goods manufacturing enterprises. (2) A total of 29.1% of the impact of servitization on enterprise performance is achieved through marketing intensity; that is, marketing intensity exerts a partial mediating effect on the impact of servitization on enterprise performance. (3) Market power play a negative regulatory role, which promotes the servitization paradox of sporting goods manufacturers but can also promote the positive impact of servitization on marketing intensity. In addition, with the expansion of market power, corporate servitization does not need to impact corporate performance through marketing activities. (4) R&D intensity negatively affects the relationship between marketing intensity and corporate performance, promotes the inhibitory effect of marketing intensity on corporate performance, and aggravates the inhibitory effect of servitization on corporate performance through marketing intensity. Based on the above conclusions, the following conclusions are drawn:

There is currently a dampening effect of servitization on the enterprise performance of sporting goods manufacturing firms. There may be an important threshold in the transformation of manufacturing into a service-oriented industry; only after the industry has crossed this inflection point can enterprises obtain a strategic resource advantage in competition [65, 79]. However, at the current stage, China’s sporting goods manufacturing listed companies are still in the early stage of service-oriented transformation, and such an inflection point has yet to be reached, which has enriched the theoretical level of the impact of servitization on different manufacturing industries. The subsequent study of the relationship between servitization and enterprise performance provides a richer reference basis. Due to the different development maturity levels of the listed sporting goods manufacturing enterprises in China, the motivations, concepts and methods of servitization differ. For small and medium-sized enterprises, which compose the main body of China’s sporting goods manufacturing industry, a "passive servicing" phenomenon can occur due to an inaccurate market assessment; that is, the original development concept and core competitiveness may become diluted. In the market heat under the call to explore the service side of a business, the firm’s technology, management and marketing capabilities and market demand may not match the situation. In this case, there is a mismatch between actual technical, management and marketing capabilities and market demand. it is necessary for enterprise decision makers to understand the stage of the enterprise life cycle and the characteristics of industry customer demand and to extend the service business depending on access to adequate resources. The integration of online and offline sales and services and the use of modern logistics construction to drive the depth of that integration, assists in the establishment of mature product brands, sales channels, customer consumption and activities. We analyze user preferences, concepts and consumption characteristics to develop effective service marketing programs. In the era of the internet, sports manufacturing enterprise services can use the digital community to penetrate the daily life of consumers and provide accurate access to consumer sports life data. An enterprise can support consumer lifestyles in product service and thus improve customer service quality perceptions and brand loyalty [80]. Moreover, the choice of servitization path, that is, upstream industry chain servitization (R&D, design) or downstream industry chain servitization (distribution, logistics and after-sales), is inseparable from the level of enterprise decision-making and management combined with the characteristics of the enterprise and the development approach of prudent determination and implementation.

In addition, due to the regular prevention and control of the COVID-19 epidemic in recent years, sporting events and other activities have been strongly affected, which has had a greater impact on both the service business revenue and the total operating income of sporting goods manufacturing enterprises engaged in event services. For example, the service business income of Jinling Sports is mainly for the equipment service of large-scale events; however, due to the tense situation under epidemic prevention and control, more events were canceled or postponed, and the company’s event service income in 2022 fell sharply by 95.53% compared with that in the previous year, accounting for only 0.45% of the proportion of operating income in that year. Although the sales of track and field equipment increased by 48%, the company’s overall turnover still fell by 13.3%. Moreover, in the case of the substantial control of business costs, the gross profit margin of the corporate event services business from 2021–2022 still has an average annual rate of approximately 18% decrease, and the overall gross profit margin of the enterprise is also undergoing a year-on-year decline. The service business of Cnsg Holdings is mainly designed to provide products, materials, special construction and all kinds of consulting services and facility operation services for event race meetings. Under the influence of the COVID-19 epidemic, enterprises have tried their best to control the cost of service business; however, in 2020–2021, the gross profit margin of the enterprise’s equipment leasing business was still declining at an average rate of 7% by the end of the research period, and in 2022, the gross profit margin was only recoverable after the cost of service business was reduced by 43%. It can be seen that in the face of the impact of the epidemic, relevant enterprises significantly reduced the cost of their service businesses but have also faced difficulty resisting the overall decline in enterprise performance, and the cost of a substantial reduction cannot guarantee the quality of service products. Service transformation has also reached a stalemate. However, with the adjustment of China’s policy on the COVID-19 epidemic pneumonia in 2023 and sporting events operations back on track, the transformation of China’s sporting goods enterprise service-oriented transformation is entering a new stage of improvement.

(2) Marketing intensity plays a partial mediating role between servitization and enterprise performance, and, based on the analysis of enterprise financial data, these results confirm the accuracy of previous theoretical studies. That is, at present, the service-oriented path of China’s sports manufacturing enterprises is still located in the downstream industrial chain path proposed by Li et al. [7]. Sporting goods manufacturing enterprises are not to possess the support resources needed to achieve service-oriented and organizational change. Enterprises can increase investment in the downstream stage of production, advertising, marketing and marketing, transportation and other service elements to carry out service-oriented transformation, which has strong achievability and a relatively low market risk. This approach has strong realizability and relatively low market risk. Moreover, the inhibitory effect of marketing intensity on the performance of Chinese sporting goods manufacturing enterprises reminds business operators to focus on continuity in the process of investing in marketing resources so that the corporate brand image in the minds of consumers is deeply rooted in the enhancement of product reputation. The optimal value of enterprise marketing investment matches that of service business investment, and an enterprise should formulate a reasonable marketing strategy to avoid the phenomenon of excessive marketing in the process of service-oriented transformation. On the other hand, market power contributes to the servitization paradox of sporting goods manufacturers, but it can positively affect the relationship between servitization and marketing intensity. The main purpose of manufacturing servitization is to obtain a differentiated competitive advantage, and unmarketability urges enterprises to win monopoly positions in imperfect competitive markets by developing their own unique resources and, in turn, obtaining monopoly rents. The servitization of manufacturing enterprises increases the cost of infiltrating the product market for competitors, thus increasing their difficult. In addition, the promotion of market power allows enterprises to have a stable source of customers and ensures the smooth flow of marketing channels; on the other hand, customers involved in enterprise servitization are also more inclined to adopt service-oriented products from enterprises with leading product technology and large market shares to ensure a good profit space. Therefore, sporting goods manufacturing enterprise servitization should further address market barriers, establish a benign competition mechanism, and explore new business types, new models and new paths by encouraging innovation and strengthening cooperation, accounting for the two key points of manufacturing and service, to more effectively promote the integration and coupling symbiosis of manufacturing and service industries.

(3) At present, China’s sporting goods manufacturing industry servitization and R&D endeavors have not yet formed a benign coupling mechanism. The technological content of servitization products needs to be improved, while the growth of R&D investment crowds out marketing funds, and small and medium-sized enterprises have created a financial burden. Therefore, local governments can introduce advanced digital service platforms to reduce the technological threshold of enterprise service-oriented products. At the same time, research results incubation centers were established, intellectual property rights and patent protection were strengthened, and key projects, key enterprises and other policies were introduced to promote the transformation of existing research results. Traditional sporting goods manufacturing enterprises should be encouraged to implement internet-based C2B (personalized customization), O2O (online and offline), B2C (direct sales) and other business model innovations. Second, enterprises should accurately locate customer groups and pay attention to consumer experience feedback. Some consumers are highly creative and have sufficient expertise and passion to participate in collaborative innovation activities, especially when sporting goods manufacturing companies take the initiative to approach and interact with members of the online virtual community who are willing to share their creativity and ideas [81], which also provides companies with product innovation with specific ideas. Finally, sporting goods manufacturing enterprises should comply with the development trend of the manufacturing industry assisted by favorable policy support to further strengthen school-enterprise cooperation, improve the construction of school-enterprise cooperation platforms, and enhance the effectiveness of the transformation of scientific and technological achievements among colleges and universities. Moreover, the introduction of high-end composite talent and training efforts to increase the number of high-end talent inputs should be strengthened, and the multilevel, a multistructured talent training layout for the transformation and upgrading of China’s sporting goods manufacturing industry should be gradually improved to provide solid support for talent.

## Research outlook

Based on the financial data of Chinese sporting goods manufacturing firms listed on the A-share and New Third Board, the mechanism of the effect of servitization on the performance of sporting goods manufacturing firms is verified in this paper through the use of unbalanced panel data covering the period of 2007–2022 and a moderated intermediation model, but this study still has the following limitations: (1) since unlisted firms in China are not mandated to disclose their corporate financial data, they do not all include the many small and medium-sized sporting goods manufacturing firms in the sample; (2) this study takes an aggregate view of all listed sporting goods firms as a pool, and the effects of different service product types on firm performance may be inconsistent across firms of different sizes and geographic regions. Therefore, in subsequent research, financial data from small and medium-sized sporting goods manufacturing enterprises should be obtained through questionnaires and field research to explore the effect of servitization on the performance of sporting goods manufacturing enterprises of different sizes to provide a more comprehensive and accurate response to the status quo of the transformation of servitization of sporting goods manufacturing enterprises in China. In addition, we further enrich the heterogeneity study of different enterprise characteristics to explore the performance of service-oriented transformation in enterprises with different life cycles and production factor types. In addition, the service-oriented types of enterprises are separated, and their impacts on enterprise performance are independently explored.
